# Supplementary material for: The Nuclear Speckles Protein SRRM2 Is Exposed on the Surface of Cancer Cells
Source: Cells. 2024 Sep 17;13(18):1563. doi: 10.3390/cells13181563 (PMC11430694; doi:10.3390/cells13181563)
Supplement: Supplementary file 1 [file cells-13-01563-s001.zip › cells-3082645-supplementary.pdf]

The nuclear speckles protein SRRM2 is exposed on the surface of cancer cells

Supplementary Figures

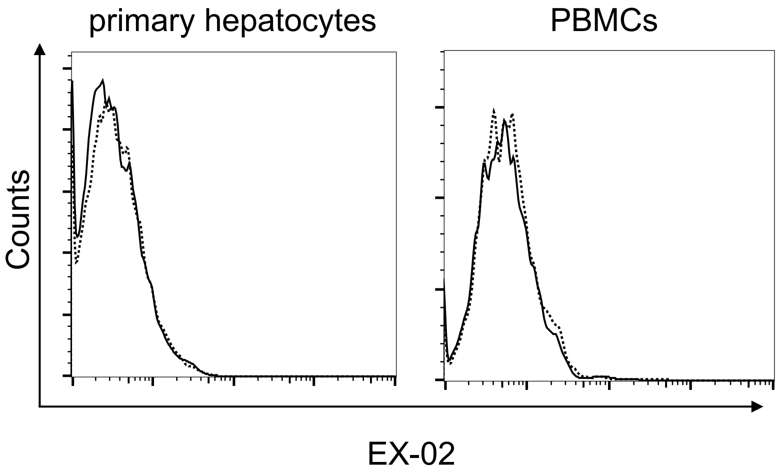

Figure S1: primary hepatocytes and PBMCs stained with EX-02 and a anti-rat IgG / Alexa 647 secondary antibody. Dotted line = isotype control antibody

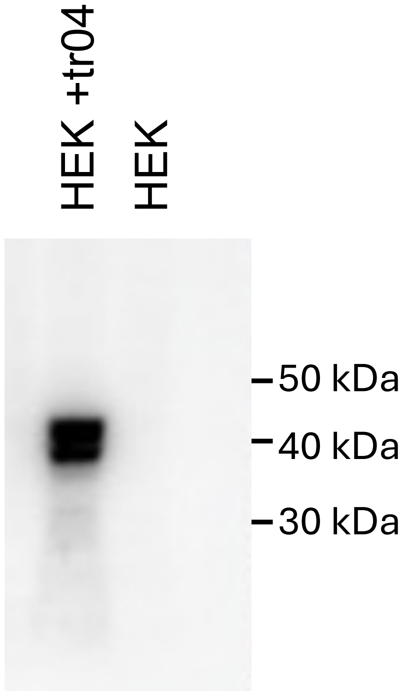

Figure S2: EX-02 immunoblot. HEK293 cells were transfected with an expression plasmid encoding a tr04-his fusion protein. Lysates from transfected and non-transfected cells were separated by PAGE, blotted, and incubated with EX-02, followed by incubation with a HRP-coupled anti-rat IgG secondary antibody and finally developed with ECL.

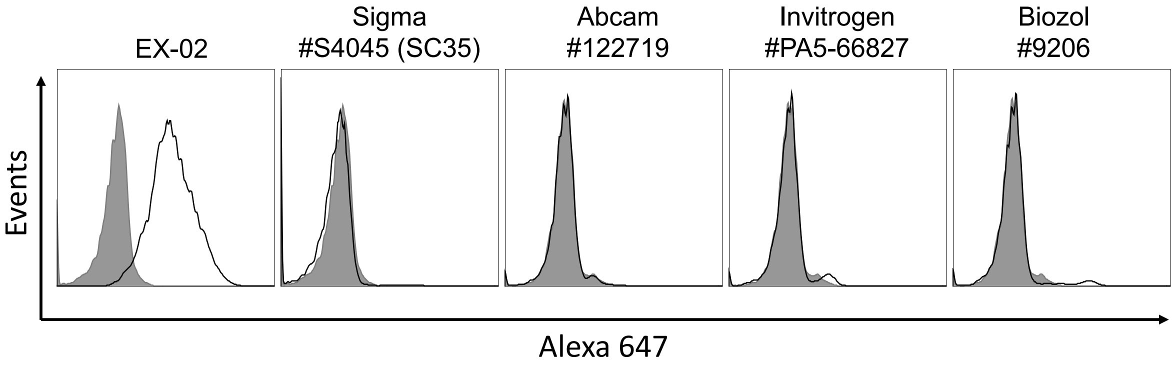

Figure S3A: HeLa cells were incubated with EX-02 or commercial SRRM2 antibodies, washed, and then incubated with suitable Alexa647-labeled secondary antibodies. Binding was measured by flow cytometry. SRRM2 = black line; isotype control = tinted grey histogram.

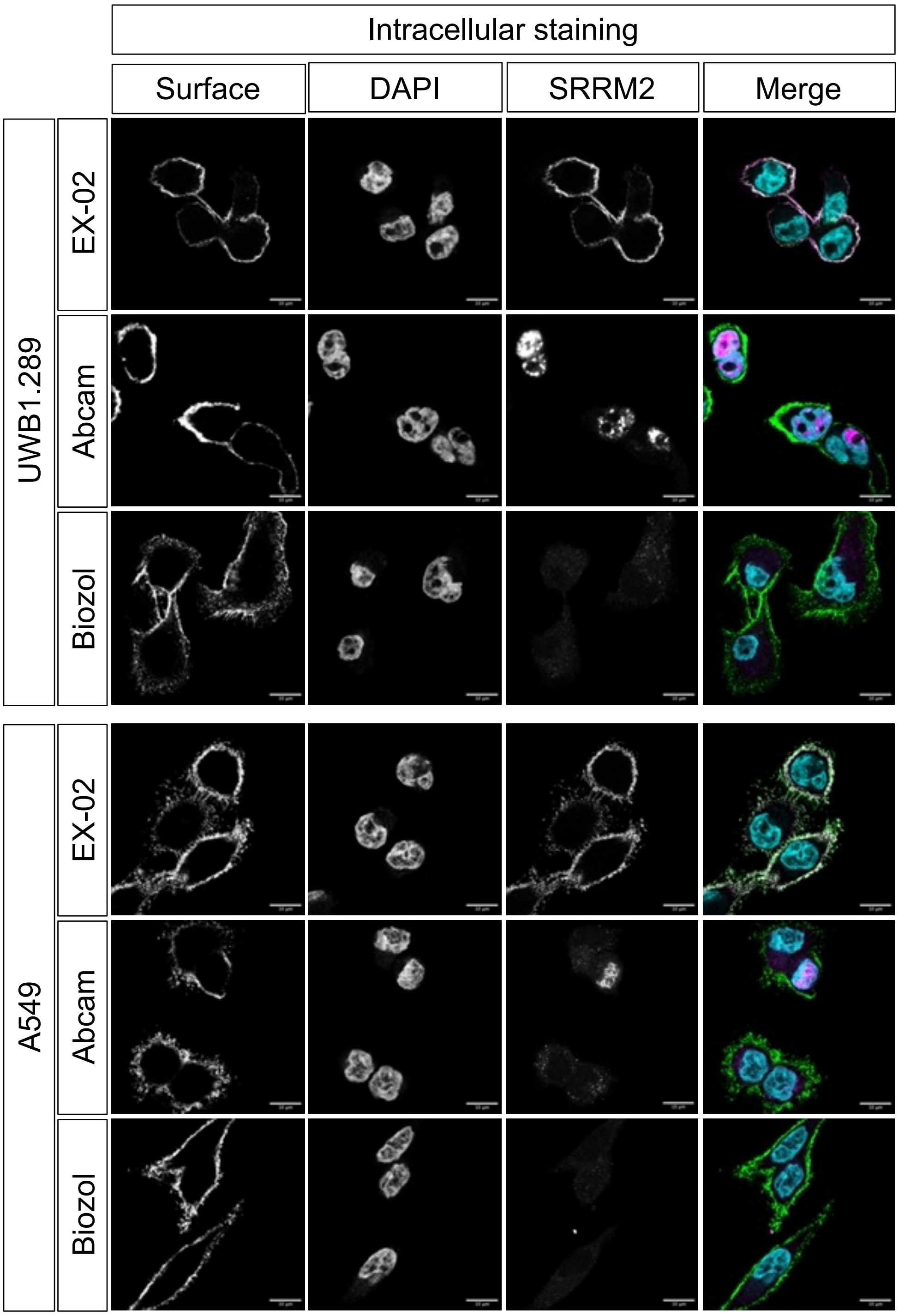

Figure S3B: Confocal microscopy with (A) vital and (B) fixed and permeabilized UWB1.289 and A549 cells. EX-02, Abcam, clone #122719 and Biozol, (MBS9609206) were used as SRRM2 antibodies (magenta), an EpCAM or IGF-α3 antibody were used to stain an established surface protein (green). Nuclei were counterstained with DAPI (cyan).

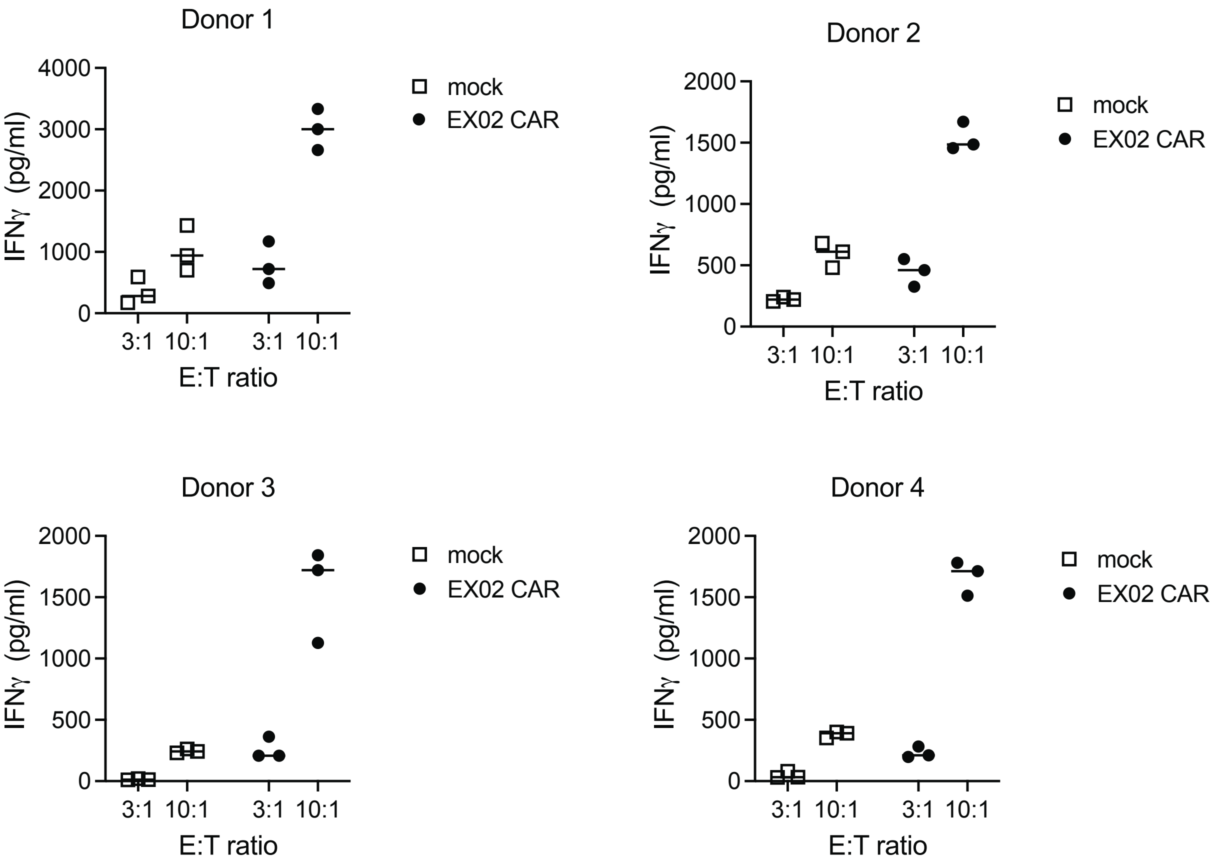

Figure S4: EX02 CAR-T cells or mock-T cells obtained from four different donors were incubated with surface SRRM2 positive PCI-1 head and neck cancer cells at different rat ios for 48 h. Secretion of IFN-ɣ into the supernatant was quantified with a com mercial sandwich ELISA.

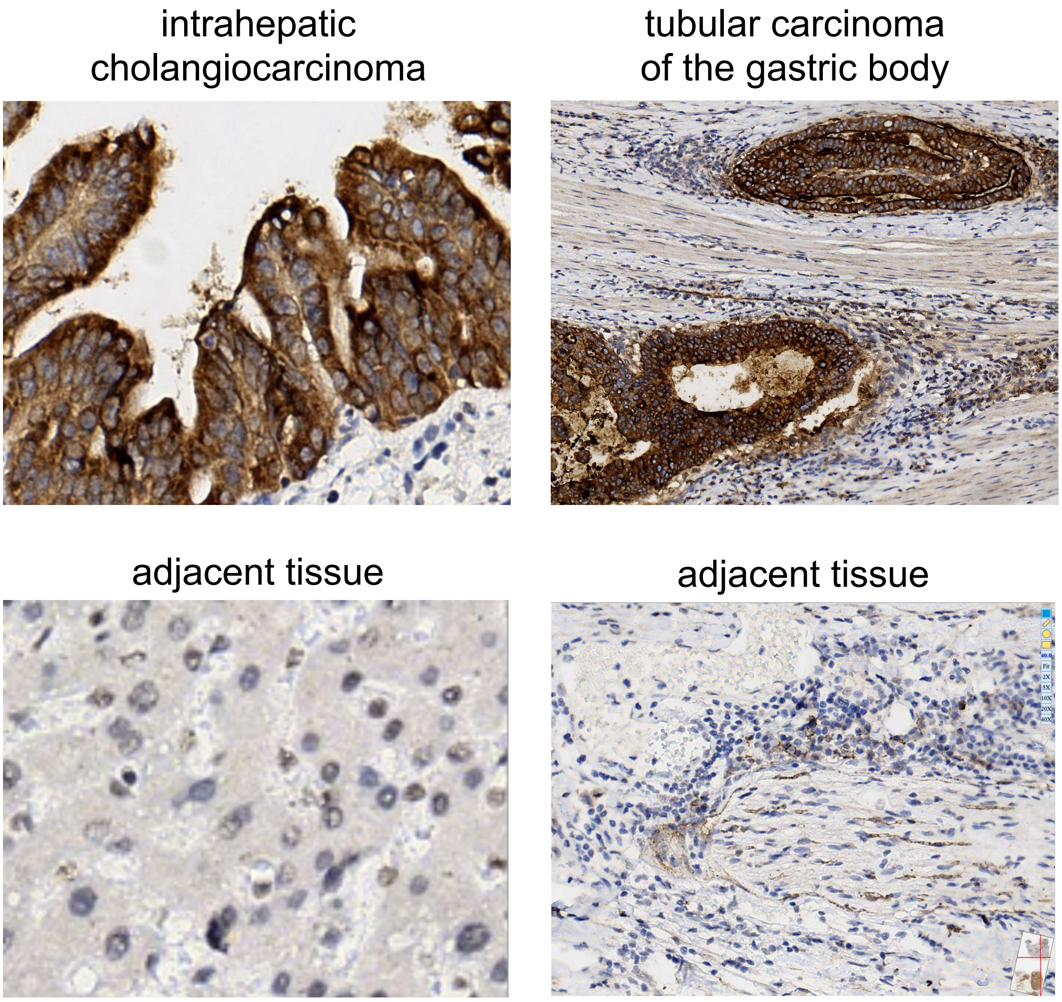

Figure S5: upper panel: EX-02 membrane staining in an intrahepatic cholangi ocarcinoma (left) and a tubular carcinoma of the gastric body (right), while nuclei stain almost completely negative. Lower panel: staining of adjacent non-cancerous t issues reveals weak nuclear staining.

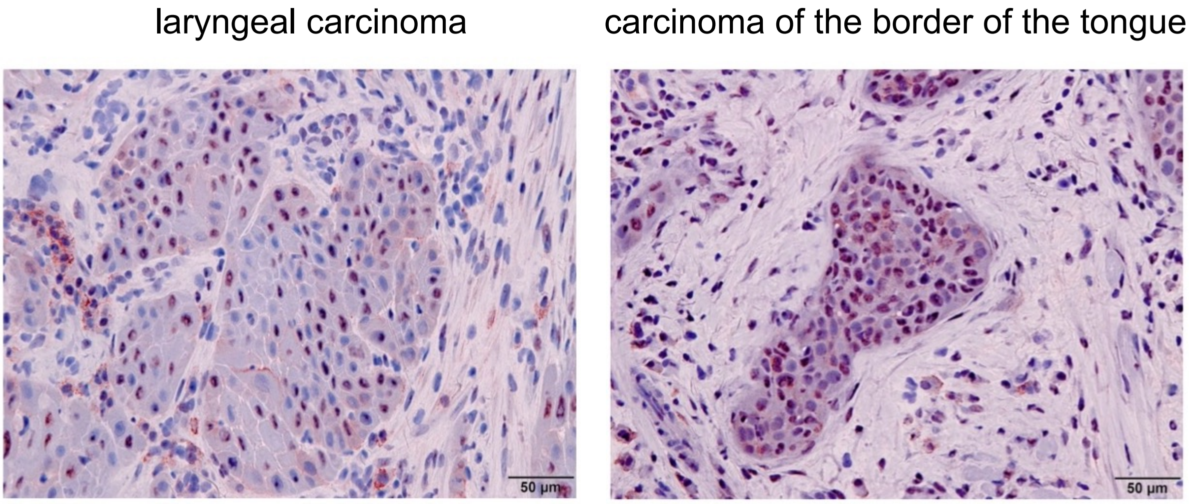

Figure S6: EX-02 staining of two different squamous cell carcinoma of the larynx (left) and the border of the tongue (right) reveals nuclear localization of SRRM2 in cancer and non-cancer cells, while surface localization is not detectable.

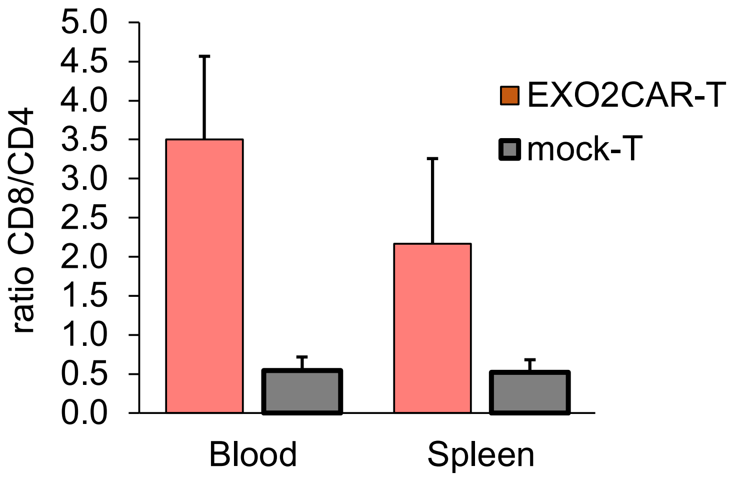

Figure S7: Mice treated with EX-02CAR-T cells also revealed a significant higher $CD8^+/CD4^+$ ratio in the blood and the spleen as compared to mock-T treated animals,
